# Why and how might genetic and phylogenetic diversity be reflected in the identification of key biodiversity areas?

**DOI:** 10.1098/rstb.2014.0019

**Published:** 2015-02-19

**Authors:** T. M. Brooks, A. Cuttelod, D. P. Faith, J. Garcia-Moreno, P. Langhammer, S. Pérez-Espona

**Affiliations:** 1 IUCN, 28 rue Mauverney, Gland 1196, Switzerland; 2 IUCN, 219c Huntingdon Road, Cambridge CB3 0DL, UK; 3 Australian Museum, 6 College St., Sydney, New South Wales 2010, Australia; 4 Het Haam 16, Arnhem 6846 KW, The Netherlands; 5 School of Life Sciences, Arizona State University, PO Box 874601, Tempe, AZ 85287-4601, USA; 6 Department of Life Sciences, Anglia Ruskin University, East Road, Cambridge CB1 1PT, UK

**Keywords:** key biodiversity areas, genetic diversity, phylogenetic diversity, adaptive potential, evolutionary refugia

## Abstract

‘Key biodiversity areas' are defined as sites contributing significantly to the global persistence of biodiversity. The identification of these sites builds from existing approaches based on measures of species and ecosystem diversity and process. Here, we therefore build from the work of Sgró *et al.* (2011 *Evol. Appl.* **4**, 326–337. (doi:10.1111/j.1752-4571.2010.00157.x)) to extend a framework for how components of genetic diversity might be considered in the identification of key biodiversity areas. We make three recommendations to inform the ongoing process of consolidating a key biodiversity areas standard: (i) thresholds for the threatened species criterion currently consider a site's share of a threatened species' population; expand these to include the proportion of the species' genetic diversity unique to a site; (ii) expand criterion for ‘threatened species' to consider ‘threatened taxa’ and (iii) expand the centre of endemism criterion to identify as key biodiversity areas those sites holding a threshold proportion of the compositional or phylogenetic diversity of species (within a taxonomic group) whose restricted ranges collectively define a centre of endemism. We also recommend consideration of occurrence of EDGE species (i.e. threatened phylogenetic diversity) in key biodiversity areas to prioritize species-specific conservation actions among sites.

## 1. Introduction

Two notable features characterize life on Earth in the twenty-first century: its distribution around the planet's surface is highly uneven and its diversity is declining fast [1]. Given this, many sectors of society demand information on the places which make disproportionate contributions to the persistence of biodiversity. Maybe most notable among these is the Convention on Biological Diversity, for which the 193 Parties have established a target to protect ‘at least 17 per cent of terrestrial and inland water, and 10 per cent of coastal and marine areas, especially areas of particular importance for biodiversity … ’ by 2020 [2,3]. However, this demand spans sectors of society including other international conventions, national and local government, the multilateral financial institutions, the private sector, and local and indigenous communities [4].

In response to this demand, numerous systems have been developed for identifying important sites for biodiversity for different taxonomic groups, different ecosystems, and different countries and regions. The earliest such efforts were initiated by what is now the BirdLife International partnership, which developed standards for the identification of Important Bird Areas in the late 1970s [5], and has now applied these in each of the world's countries to identify more than 10 000 sites globally [6]. Similar approaches have been developed for the identification of Important Plant Areas [7], Prime Butterfly Areas [8], Alliance for Zero Extinction sites [9], ‘B-ranked’ sites in North America [10] and key biodiversity areas in freshwater [11] and marine [12] ecosystems, among others. These approaches have used four types of criteria to trigger site identification, based on the presence in the site of threshold quantities of (A) threatened species, (B) restricted-range species assemblages or centres of endemism, (C) species characteristic of a particular ecosystem or (D) species congregations and aggregations.

However, while these taxon-, ecosystem- and theme-specific approaches to the identification of important sites have delivered substantial benefits by providing information on where site safeguard can make the greatest contributions towards reducing the rate of biodiversity loss [6], the proliferation of such approaches has also generated duplication of effort and policy confusion. In response to this, the approximately 200 government and government agencies and approximately 1000 non-governmental organizations that comprise the membership of the International Union for the Conservation of Nature (IUCN) passed a resolution at the 2004 World Conservation Congress in Bangkok which ‘requests the SSC, working in partnership with IUCN members, to convene a worldwide consultative process to agree a methodology to enable countries to identify Key Biodiversity Areas, drawing on data from the IUCN Red List of Threatened Species and other datasets, building on existing approaches'.

Over the last decade, the IUCN Species Survival Commission and World Commission on Protected Areas have been jointly leading this process. Building from an initial scientific paper [13], constructive debate in the scientific literature [14,15] and best practice guidelines [4], the two Commissions convened a ‘framing workshop’ in Cambridge in June 2012. This workshop, bringing together 66 participants (from 52 organizations across 19 countries) from across science, policy and practice, forged a common definition of key biodiversity areas: sites contributing significantly to the global persistence of biodiversity.

One implication of this definition is that it considers biodiversity comprehensively, in contrast to the focus of existing approaches on specific taxa or ecosystems. Thus, a challenge for the process of consolidating a global standard for the identification of key biodiversity areas has been to ensure that the criteria for site identification span scales and components of ecological organization. Noss [16] derived a scientific definition for biodiversity as spanning genetic and population, species and ecosystem levels of ecological organization, and comprising compositional, structural and functional components. The Convention on Biological Diversity [17] formalized this definition by stating that ‘Biological diversity means the variability among living organisms from all sources including, inter alia, terrestrial, marine and other aquatic ecosystems and the ecological complexes of which they are part: this includes diversity within species, between species and of ecosystems'.

Here, we develop a general framework and three specific recommendations on how the process of consolidating a global standard for the identification of key biodiversity areas might address components of biodiversity below the species level, for consideration in the review of the draft standard. These include genetic diversity within species, phylogenetic diversity among species and the evolutionary processes which drive and maintain both of these [18]. This paper draws not only from the key biodiversity area framing workshop mentioned above, but also from subsequent workshops addressing key biodiversity area criteria (Front Royal, March 2013) and thresholds (Rome, December 2013), as well as from discussion at meetings of ConGRESS (Gregynog, April 2013) and of the Royal Society (London, March 2014).

## 2. A framework for addressing biodiversity below the species level in key biodiversity area criteria

The many ways in which biodiversity below the species level could be addressed in the key biodiversity area criteria can be organized through the framework presented by Sgrò *et al.* [19], originally intended for consideration of ‘evolutionary resilience’ in climate change response strategies. Here, we generalize this to guide the incorporation of genetic and phylogenetic biodiversity into the process of informing decision-making more broadly, and align it to Noss's [16] and Gaston's [20] definitions of biodiversity to structure our consideration of biodiversity below the species level in key biodiversity area criteria (table 1).

**Table 1.** Framework for consideration of genetic and phylogenetic diversity relative to the criteria for identification of key biodiversity areas.

aim [19]	biodiversity component [16]	key biodiversity area criterion
increase population size and genetic variation generally	genetic and population structure and composition	threatened species
maintain adaptive potential in target genes and traits	genetic and population structure and function	
identify species with little adaptive potential		
identify and protect evolutionary refugia	genetic and population structure and composition	centres of endemism
increase connectedness and gene flow across environmental gradients	genetic and population structure and function	n.a.
increase adaptability to future environments by translocation	genetic and population structure, function and composition	

The relevance of the last two elements of the Sgrò *et al.* [19] framework lies beyond the identification of key biodiversity areas *per se*. To ‘increase connectedness and gene flow across environmental gradients' generally requires planning and action at the levels of entire landscapes or seascapes—beyond individual sites. This is the case even where environmental gradients are very sharp (e.g. in many mountain and coastal ecosystems), and certainly where they are represented by broad ecotones. For example, Smith *et al.* [21] found high morphological divergence between those Cameroonian *Andropadus virens* populations in tropical forest and those in forest-savannah, compared with low divergence (for the same level of gene flow) between tropical forest sites and between forest-savannah sites. There is some counter-evidence against this: Henry *et al.* [22] found low dispersal along an elevational gradient by American pikas *Ochotona princeps* in British Colombia. In any case, the location of given key biodiversity areas along environmental gradients will be relevant in prioritizing their conservation and connectedness, where these are important for the persistence of the biodiversity feature triggering the site's identification [23].

Meanwhile, given that key biodiversity areas are sites contributing significantly to the persistence of biodiversity features which they currently hold, to ‘increase adaptability to future environments by translocation’ (along with other aspects of relocation including species reintroduction and ecosystem restoration) is not relevant *a priori* to the identification of key biodiversity areas. However, translocation (or reintroduction or restoration) could trigger the identification of new key biodiversity areas, if, as, and when such actions are successful enough to trigger the criteria for a new site in their own right [24].

We also considered whether there might be any aims for incorporation of biodiversity below the species level in informing decision-making more broadly which were not proposed in Sgrò *et al*.'s [19] framework, and which we should therefore add to table 1, but were not able to identify any such omissions.

We discuss each of the elements in turn, seeking to develop practical recommendations of how consolidating a global standard for the identification of key biodiversity areas might address components of biodiversity below the species level. In each case, we strive to strike a balance in how demanding we are of genetic and phylogenetic data availability. Thus, on the one hand, our proposals should be robust enough to be applied today in conditions of relatively sparse data availability, while on the other hand, they should be unlikely to destabilize site identification (e.g. through identification of orders of magnitude more sites), if, as, and when data volumes increase into the future.

## 3. Genetic diversity

The first component of Sgrò *et al*.'s [19] framework recognizes the great genetic variation within individual species. Intraspecific genetic variation is structured across a large range of variation depending on issues such as the species' historical dynamics and demography, and topography. For example, many species show genetic homogeneity within certain boundaries and exhibit a marked difference from conspecifics beyond those boundaries. Other species show genetic variation that is well correlated with distance, with genetic differences increasing or decreasing proportionately with the physical distance separating the populations being sampled. Regardless of the actual structuring of the intraspecific variation, it is generally agreed that geography plays an important role [25–27] and that this is important for conservation prioritization and other applications [28]. This is apparent with the recent proliferation of studies on landscape genetics [29], assessing the effect of landscape features on gene flow and genetic diversity by combining genetics, GIS techniques and spatial statistics [30–32].

Given this, an argument can be constructed for ensuring that the criterion for presence of threshold populations of a threatened species triggering key biodiversity area identification be extended to also consider ‘threatened genetic diversity’. Thus, for criteria structured in the form of ‘at least X% of the global population of a threatened species occur at a site’, this population metric could be supplemented by one of ‘X% genetic diversity of a threatened species being unique to the site’. This would ensure that sites holding a disproportionately high genetic diversity of a threatened species triggered key biodiversity area identification, even if the population of the species at the site was relatively small and insufficient to trigger site identification in its own right. The use of a subcriterion requiring the presence of a threshold population of ‘functional reproductive units' at the site would ensure that this extension does not trigger identification of sites holding tiny, unviable populations.

*Recommendation no. 1*. Thresholds for the threatened species criterion currently consider a site's share of a threatened species' population; expand these to include the proportion of the species' genetic diversity unique to a site.

While this recommendation would ensure that key biodiversity areas are indeed sites contributing significantly to the persistence of genetic diversity overall, these sites may be very different from those where genetic diversity contributes significantly to the evolution or persistence of the species in question. Thus, Petit *et al.* [33], for example, found that while centres of genetic diversity for 22 species of European trees are concentrated in central Europe, centres of diversification are mainly Mediterranean; Vandergast *et al.* [34] found similar differences for 21 vertebrate and invertebrate species in California. This is because nearly all variation in genomes, from butterflies [35] to humans [36], is neutral—only a small fraction is adaptive [37]. Although markers assessing adaptive variation might sometimes produce similar patterns as neutral markers [38], we cannot assume that neutral variation is a surrogate for adaptive markers [39]. Sgrò *et al.* [19] recognized the importance of assessing adaptive genetic variation as the second and third components of their framework.

The general approach to addressing adaptive variation in practice has been to incorporate it into the definition of evolutionary significant units [40–42]. The rapid acceleration of technology in the field of genomics is allowing the development of new methods for comprehensive evaluation of adaptive diversity [43], although in the medium-term it is unlikely that we will see studies incorporate adaptive loci to the already widespread use of neutral loci for large numbers of species. While the application of genomic tools is still in its infancy, these offer a great opportunity for genetic marker discovery and the study of adaptive genetic loci on a wide range of species [44,45], and metagenomics approaches could facilitate the identification of key biodiversity areas [46].

In the meantime, a rule of thumb for incorporating adaptive genetic diversity into the identification of key biodiversity areas could be simply to broaden the scope of the threatened species criterion to consider ‘threatened taxa’, as long as these are globally relevant. The IUCN Red List of Threatened Species [47], which provides the units for evaluation under this criterion, is robust to application not only at the species level but also at the level of subspecies, plant varieties (e.g. forma, morph and cultivar), and isolated subpopulations [48]. Given that the Red List does not assess non-threatened taxa below the species level, this rule of thumb would not be extended to the other species-level criteria for the identification of key biodiversity areas (i.e. restricted-range species, aggregations). Fjeldså's [49] analyses of Andean birds suggest that such a modification may make little difference to which sites are triggered as key biodiversity areas, but others have anticipated that the recognition of infraspecific taxa would identify additional sites of significance for birds in Mexico [50] and the Philippines [51].

*Recommendation no. 2.* Expand criterion for ‘threatened species' to consider ‘threatened taxa’.

While such a recommendation would in effect allow this criterion to support the conditions for future diversification, it is not framed as such given the very slow rate of macro-evolution relative to anthropogenic land-use impacts. The fastest documented rates of speciation are for *Lupinus* in the Andes [52], *Laupala* crickets in Hawaii [53], and for fish species in Lake Victoria and Lake Malawi [54]. However, the latter have unfolded over the last approximately 15 000 years—at least three orders of magnitude slower than the timescale of human land-use decision-making.

## 4. Phylogenetic diversity

The fourth component of Sgrò *et al.*'s [19] framework recognizes the importance of evolutionary refugia, where geographical isolation has allowed speciation across multiple taxonomic groups through drift. The significance of such sites for the global persistence of biodiversity is therefore their contributions to the maintenance of this unique evolutionary history [55–57].

The last two decades have seen substantial advances in the compilation of phylogenies from molecular and other types of data, and their calibration to derive trees that incorporate time into their branch lengths using molecular clock approaches [58]. These have allowed the development of methods for measuring the unique contributions of specific places to phylogenetic diversity [59,60], as well as the calculation of continuous surfaces of phylogenetic endemism [61]. It remains unclear whether optimal selection of such sites identifies places which are different from [62–64] or similar to [65–67] those identified based on species endemism. However, even if only a few sites important as evolutionary refugia do not emerge as centres of species endemism, it is valuable to incorporate a mechanism by which the criteria for identification of key biodiversity areas can address these [68].

The general form for key biodiversity area criteria for centres of endemism can be expressed as requiring that a site holds ‘at least X% of the species within a taxonomic group whose restricted ranges collectively define a centre of endemism’. This criterion encompasses three operational components: (i) the definition of the taxonomic group for consideration (typically class for vertebrates, order for other taxa, based on practical applicability); (ii) the definition of a maximum range size for species whose overlapping ranges can define a ‘centre of endemism’—Stattersfield *et al.* [69] used 50 000 km^2^, corresponding to the 25th percentile of the range-size distribution in the class Aves, and a minimum of two species to define an Endemic Bird Area; and (iii) the definition of the proportion of these restricted-range species necessary to confirm the site's identification as a key biodiversity area.

The second and third of these components could be extended to consider evolutionary refugia based on a site's complement of the phylogenetic diversity restricted to the centre of endemism [60]. Such an approach would extend the criterion requiring that a site holds ‘at least X% of the complement of species within a taxonomic group whose restricted ranges collectively define a centre of endemism’ to require, more generally, that a key biodiversity area hold ‘at least X% of the compositional or phylogenetic diversity of species within a taxonomic group whose restricted ranges collectively define a centre of endemism’.

*Recommendation no. 3*. Expand the centre of endemism criterion to identify as key biodiversity areas those sites holding a threshold proportion of the compositional or phylogenetic diversity of species (within a taxonomic group) whose restricted ranges collectively define a centre of endemism.

## 5. Applications to species-specific conservation

While the definition of key biodiversity areas recommended by the framing workshop establishes them as important sites for biodiversity, this does not imply any particular kind of conservation management action upon them (although many may require them, and many may already be under some management regime). This said, knowledge of where key biodiversity areas are and what biodiversity features trigger them is used by many different sectors of society for many different kinds of applications. These include conservation actions; the science of optimal allocation of resources for such conservation actions is known as systematic conservation planning [70,71]. Most frequently, these actions relate to site safeguard and management, for which the planning process is known as spatial conservation prioritization [71]. However, key biodiversity area information is also useful in application to other aspects of conservation action, including single-species management.

Bearing this in mind, one aspect of evolutionary history notable by its absence from the four recommendations above is the incorporation of the phylogenetic diversity unique to a species (its ‘unique PD contribution’; [60]) into the criteria for key biodiversity area identification. A number of such measures exist, based for example on the time since divergence from a species' closest relative, and are used in combination with information on the species' extinction risk to set priorities for species conservation [72–77]. The Zoological Society of London uses this approach, for example, to guide their Evolutionarily Distinct and Globally Endangered (EDGE) programme [68,78].

Safi *et al.* [79] combined the EDGE approach with coarse resolution species range maps to guide prioritization of conservation actions among broad regions. Faith [68] proposes a modified approach which calculates the loss in threatened branches or phylogenetic diversity if the area were lost, addressing the challenge that multiple EDGE species in an area can represent a large or a small amount of threatened phylogenetic diversity, depending on whether the species are phylogenetically clumped or dispersed. Given that all EDGE species are by definition globally threatened, all in turn will have those sites contributing significantly to their persistence identified as key biodiversity areas (under the criterion for ‘threatened taxa’, see above). One interesting application of key biodiversity area information could therefore be to refine the approaches proposed by Safi *et al.* [79] and combine them with site-specific measures such as threats to the species and costs of ameliorating these threats to prioritize specific sites for actions to conserve EDGE species specifically [78] and threatened phylogenetic diversity generally [68].

*Recommendation no. 4*. Consider occurrence of species making the greatest contributions to maintaining phylogenetic diversity (i.e. EDGE species) in key biodiversity areas to inform prioritization of species-specific conservation actions among sites.

## 6. Conclusion

The initial discussions of the IUCN Species Survival Commission and World Commission on Protected Areas process considered establishment of a new subcriterion for triggering key biodiversity area identification for sites contributing significantly to the global persistence of biodiversity through their importance in maintaining outstanding evolutionary process, possibly to sit alongside the criteria for outstanding ecological process (e.g. species congregations and aggregations). However, in subsequent deliberations, it proved impossible to establish a mechanism by which such a criterion could be put into operation. One possible final recommendation would therefore be to establish a non-operational criterion for sites of global significance for evolutionary process and leave the operationalization of this as a priority for future research.

On reflection, the development of such a criterion appears to be unnecessary in any case. Our three recommendations for incorporation of components of biodiversity below the species level into existing key biodiversity area criteria appear to span aspects of composition, structure and function of genetic diversity, and also the breadth of mechanisms for putting this into practice proposed by Sgrò *et al.* [19]. Much work remains for developing specific guidance for how these recommendations should be applied in practice, and overcome data limitations to allow them to be implemented for more than a handful of well-studied species. These will doubtless evolve over coming years as the power and accessibility of genetic and genomic techniques continues to improve. Nevertheless, we believe that implementation of the recommendations proposed here for key biodiversity area identification would allow confidence in the claim that such sites do indeed contribute significantly to the global persistence not just of species and ecosystem components of biodiversity but of genetic components as well.
